# The Management of Radiation-Induced Sarcomas: A Cohort Analysis from a Sarcoma Tertiary Center

**DOI:** 10.3390/jcm10040694

**Published:** 2021-02-10

**Authors:** Mateusz Jacek Spałek, Anna Małgorzata Czarnecka, Piotr Rutkowski

**Affiliations:** 1 Department of Soft Tissue/Bone Sarcoma and Melanoma, Maria Sklodowska-Curie National Research Institute of Oncology, 02-781 Warsaw, Poland; anna.czarnecka@gmail.com (A.M.C.); piotr.rutkowski@pib-nio.pl (P.R.); 2 Department of Experimental Pharmacology, Mossakowski Medical Research Centre, Polish Academy of Sciences, 02-106 Warsaw, Poland

**Keywords:** radiation-induced neoplasms, sarcoma, rare diseases, radiotherapy, cancer survivors, radiation effects

## Abstract

(1) Background: Radiation-induced sarcomas (RIS) are rare diseases with poor prognoses. The aim of the study was to analyze outcomes and identify factors affecting survival in a cohort of patients with RIS. (2) Methods: We included consecutive patients with RIS that we found in the available electronic medical records of a sarcoma tertiary center. We analyzed patients’ RIS characteristics, management of RIS, the occurrence of local recurrence and distant metastases, the date of disease progression, the date of death, and the date of the last follow-up. (3) Results: Fifty-eight patients met the inclusion criteria. The most frequent sites of RIS development were the thorax and pelvis. The majority of RIS were poorly differentiated, high-grade tumors. Forty patients underwent surgery or radiotherapy with curative intent. The others were referred to palliative chemotherapy. Median progression-free survival and overall survival were 15 and 21 months, respectively. Treatment with curative intent and tumor localization on breasts and upper extremities were associated with a lower risk of death in univariate analysis. (4) Conclusions: The study confirms the poor prognosis of RIS. Treatments with locally curative intent at the tumor site are of prognostic value. Secondary radiotherapy is rarely used in RIS.

## 1. Introduction

Radiation-induced sarcomas (RIS) are iatrogenic malignancies developing after irradiation because of previous cancer or another disease treated with high-dose radiotherapy (RT) [1]. The RIS occurrence rate is 0.03–0.2% at 10 years [2]. The cumulative RIS incidence in the population of patients who received radiotherapy (RT) is 3.2 per 1000 at 15 years, whereas the incidence of primary sarcomas is 2.3 per 1000 in patients who did not receive RT [3]. RIS account for three to six percent of all diagnosed sarcomas [4,5,6,7,8]. However, the incidence is increasing, which may be caused by several factors [9]. First, the reason might be better survival of patients who receive RT because of new systemic therapies, surgical techniques, and other treatment options [10]. Second, the number of indications for RT as a part of organ-sparing, conservative, or definitive treatment increased in the last 30 years; for example, it is indicated for rectal, cervical, breast, and primary soft tissue neoplasms. Third, new, dynamic RT techniques with intensity modulation provide better organs-at-risk sparing, but this occurs at the expense of exposure of larger volumes of healthy tissues to low-dose radiation [11]. That may lead to genomic instability and further malignant transformation [12].

Due to the rarity of RIS, no guidelines nor randomized prospective clinical trials on this topic exist. Thus, the management of RIS is challenging. The only curable modality in non-metastatic RIS is curative resection with wide negative margins [13]. The role of secondary RT in locally advanced RIS is unclear, mostly due to the concerns about possible severe side effects after re-irradiation. Chemotherapy and targeted therapy may be used in metastatic disease, but their role in the management of localized RIS is not established.

This retrospective study aimed to identify patient, tumor, and treatment characteristics of RIS. Additionally, we evaluated outcomes of treatments and prognostic factors.

## 2. Materials and Methods

### 2.1. Analyzed Cohort

We performed a retrospective analysis of a cohort of patients with RIS who were treated in our center between 1998 and 2019. We included consecutive patients with RIS by using modified criteria provided by Huvos et al.: (1) the patient received RT; (2) the neoplasm occurred within the RT volume; (3) a latency period had elapsed; (4) cancer predisposition syndromes such as Li-Fraumeni were excluded [14].

We performed a search for all available electronic medical records through MedStream Designer software from Transition Technologies. Corresponding International Classification of Diseases codes C40, C41, C45, C46, C47, C48, C49, and the keyword “induced” were used. The following parameters were analyzed: patients’ characteristics, site and pathological diagnosis of the primary tumor, date of primary RT, date of RIS diagnosis, RIS pathological diagnosis and grade, management of RIS, the occurrence of local recurrence and distant metastases, date of disease progression, date of death, and date of the last follow-up. All available records were reviewed independently by two co-authors. Missing data regarding the date of death, if applicable, were obtained from the National Cancer Registry.

### 2.2. Statistical Analysis

Follow-up time was calculated using the reverse Kaplan–Meier method. Progression-free survival (PFS) was calculated from the diagnosis of RIS to the last follow-up (censored), disease progression, or death. Overall survival (OS) was calculated from the diagnosis of RIS to the last follow-up (censored) or death. The Kaplan-Meier method for estimating survival functions and the Cox proportional hazards model for estimating the effects of covariates on the hazard of the occurrence of disease progression or death were used. All *p* values <0.05 were considered significant. The evaluation of data was performed using the R software environment, version 3.6.3 (R Foundation for Statistical Computing, Vienna, Austria), and the jamovi project, version 1.6.14 (retrieved from https://www.jamovi.org, Sydney, Australia).

## 3. Results

### 3.1. Patients’ Characteristics and RIS Diagnosis

Fifty-eight patients met the inclusion criteria. Forty-three of them were female, and 15 were male. Median follow-up time was 15 months (interquartile range (IQR): 9–24 months), with a maximum of 230 months. The most frequent sites of both primary cancer and RIS were the thorax and pelvis. The group comprised soft tissue sarcomas (79%) and bone sarcomas (21%). The vast majority of RIS were poorly differentiated, high-grade tumors. The most frequent pathological diagnoses were undifferentiated pleomorphic sarcoma (24%) and sarcoma not otherwise specified (21%). Table 1 and Table 2 provide a summary of primary tumor patients’ data and RIS.

### 3.2. RIS Management

**Figure 1:**
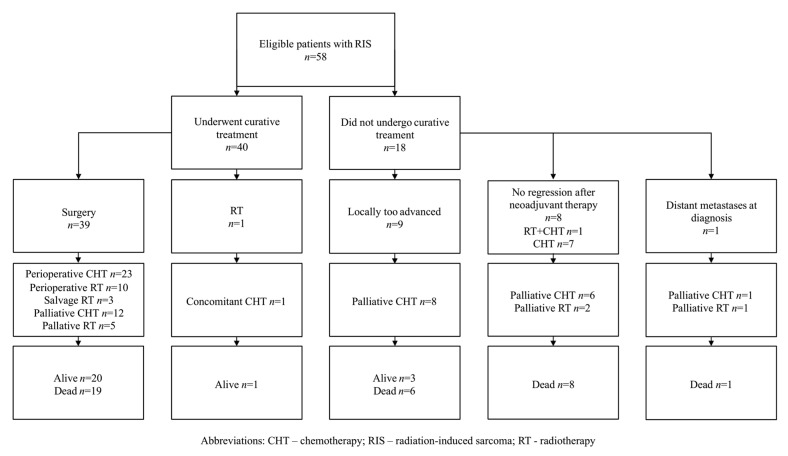
Applied methods of treatment in analyzed cohort.

Forty-eight patients were preliminarily amenable to curative treatment. The others (*n* = 10) were locally too advanced for curative treatment (*n* = 9) or had synchronous distant metastases at the moment of diagnosis of RIS (*n* = 1); all but one of them received palliative chemotherapy. Among the patients who were eligible for curative treatment, eight did not respond to induction chemotherapy (*n* = 7) or radiochemotherapy (*n* = 1) and were locally too advanced to undergo curative surgery or RT; those patients were referred to palliative treatment. One patient received curative RT combined with chemotherapy as seen in Figures S1 and S2. Thirty-nine patients underwent surgery with curative intent. Among them, R0, R1, and R2 resections were obtained in 18, 14, and 1 patient, respectively. In six cases, margin status was not available. The applied methods of treatment are summarized in Figure 1.

### 3.3. Survival Analysis

At the moment of analysis, 24 patients were alive (41%). New distant metastases were observed in 16 cases. Local recurrence after curative treatment occurred in 18 cases. Median PFS was 15 months (95% confidence interval (CI) 11–21 months). Five-year PFS was 22% (95% CI 12–42%). Median OS reached 21 months (95% CI 18–47 months). Five-year OS was 17% (95% CI 8–37%). In the univariate analysis, we found a strong influence of curative treatment to hazard ratio (HR) on death (HR 0.21, *p* < 0.001). Moreover, RIS that developed in the breasts and upper extremities were associated with a lower risk of death (HR 0.09, *p* = 0.05 and HR 0.06, *p* = 0.026, respectively) in the univariate analysis (see Table 3). Due to the relatively small number of patients (*n* = 58) and the low number of events (*n* = 33), we abandoned the multivariate analysis.

**Figure 2:**
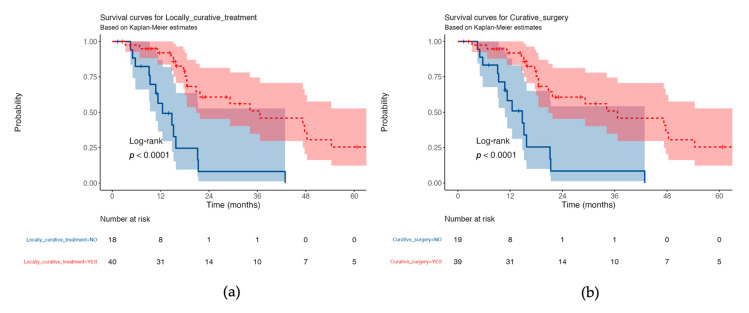
Overall survival curves with 95% confidence intervals for all enrolled patients: (**a**) locally curative treatment vs. no locally curative treatment; (**b**) curative surgery vs. no curative surgery.

Median OS differed between patients who underwent treatment with curative intent and those who did not (37 vs. 13 months, *p* < 0.0001; see Figure 2a), and between patients who underwent curative surgery and those who did not (37 vs. 15 months, *p* < 0.0001; see Figure 2b).

In the univariate analysis, we did not find an influence of surgical margins and perioperative treatment to HR on death in the subgroup of patients treated with curative surgery (see Table 4).

## 4. Discussion

RIS are very rare entities that may develop several years after primary RT. In our study, the median latent period was 11 years. RIS in our cohort presented aggressive tumor subtypes and behavior, being intermediate or high-grade in the vast majority of cases (95%). Despite diagnosis at an early or locally advanced stage and intensive treatment, results remain unsatisfactory, with a median OS as low as 21 months. The only factor strongly impacting survival is surgery with curative intent. Similar findings were reported in a recently published cohort analysis of RIS [15]. However, we did not find an influence of obtained margins and perioperative treatment on OS in a group of patients treated with curative surgery. Interestingly, perioperative chemotherapy was used more frequently than secondary RT in a previously irradiated volume (59% vs. 26%), although guidelines recommend perioperative RT in the majority of locally advanced or high-grade primary soft tissue sarcomas [16,17].

Data concerning RIS remain greatly limited. Available larger series describe RIS epidemiology or cohorts of breast cancers survivors, but only a few focus on treatment regimens [5,18,19,20,21,22,23]. All available reports highlight the poor prognosis for patients with RIS, which is in concordance with results from the current study. Importantly, in the two studies, survival rates in RIS were reported as worse than in sporadic soft tissue sarcomas [13,18].

In our study and other cohorts, the most frequent site of RIS development is the thoracic region, which is frequently irradiated due to breast cancer or Hodgkin lymphoma. Those neoplasms are associated with long life expectancy and a higher risk of RIS. The pathological pattern of thoracic RIS in our cohort confirms the high occurrence of angiosarcoma (24%), a sarcoma subtype strongly associated with the previous treatment of breast cancer [20,24]. Although it may seem like the number of radiation-induced angiosarcomas in our cohort is low, the majority of them are usually treated in breast cancer units outside sarcoma tertiary centers. Interestingly, in the current study, RIS localized in the breast and upper extremities were associated with a lower risk of death. This could be explained by the greater possibility of performing curative surgery with adequate margins, up to mastectomy or extremity amputation.

Nevertheless, pathological subtypes in the entire current cohort of patients with RIS are similar to those presented in other cohort studies with RIS that focus on results of treatment [15,19]. However, in our series, obtained surgical margins did not affect patients’ survival; whereas, in the aforementioned cohorts, gross positive resection margin was predictive for poorer survival. This discrepancy may be explained by the different approaches preferred by multidisciplinary tumor boards in sarcoma reference centers. In the current study, R2 margin was present only in one patient. In the study published by Cha et al., 32% of patients had gross positive margins [19]. In turn, in our study, 67% of patients underwent curative surgery, as compared to 90% in the aforementioned report. That may suggest distinct criteria of resectability in both institutions.

Surgery with curative intent is a mainstay of therapy. However, it may not be feasible due to anatomical location, fibrotic changes after previous irradiation, multifocal disease, or invasion of surrounding vital organs. In the current study, among patients who did not undergo surgery, nine were locally too advanced to receive such treatment and were referred to palliative chemotherapy. The other eight patients did not achieve satisfactory local response to induction treatment to undergo curative surgery. Importantly, in this combined subgroup, only one patient received preoperative RT. It has been shown that preoperative RT alone or combined with systemic treatment may provide substantial benefits to locally advanced soft tissue sarcomas [25,26,27]. RT is not frequently used in previously irradiated volumes due to the risk of significant toxicity, lack of appropriate knowledge about tolerance doses, and unknown repair of healthy tissues several years after primary RT. Additionally, there could be a strong psychological barrier related to re-irradiation. Patients may refuse secondary RT, fearing the treatment modality that caused RIS.

Moreover, RT techniques and regimens constantly evolve, and it is frequently impossible to reassess dose distribution that was delivered 20 or more years ago using other RT methods. However, RT may be carefully used in selected patients. An interesting approach could be a combination of RT with local or regional hyperthermia that enhances oxygenation and inhibits repair of RT-related damage in sarcoma cells [28,29,30]. In a retrospective cohort analysis, the authors presented the results of re-irradiation combined with hyperthermia of 16 patients with RIS in the thoracic area, which is the most frequent site of RIS development [31]. The most represented pathology was angiosarcoma (69%). In 12 patients who were eligible for assessment, the response rate was 75%, including seven complete responses and two partial responses. Only one patient developed severe late toxicity, which resulted in forearm amputation five years after treatment. Among the remaining patients, the authors described mild, late toxicities in six of them. Currently, one prospective clinical trial evaluates the combination of hypofractionated RT with hyperthermia in radiation-induced or recurrent soft tissue sarcomas (NCT04398095) [32].

This study has weaknesses. The obtained sample of patients may not be representative due to a selection bias caused by the retrospective nature of the analysis. To minimize the effect of selection, all data were reviewed by two co-authors independently (MJS, AMC). Moreover, with the retrospective nature and the presence of events that occurred decades ago, there is a significant risk of incomplete data or data misinterpretation. Additionally, the soft tissue and bone tumor classifications, available diagnostic tools, RT techniques, and treatment methods have changed in the last 30 years. Therefore, our cohort might not be representative of the contemporary population. Nevertheless, due to the long period of RIS development after RT, the aforementioned situation is inevitable. Another weakness of the study is the lack of precise RT data and dose distribution analysis, which could also be prognostic factors. RT details might have provided important data for further RT planning recommendations, especially for patients with expected long-term survival and risk of RIS development. However, the aforementioned data were poorly available or unavailable due to the long period from RT for a primary tumor or RT performed outside our center; the earliest RT in our cohort was applied in 1976. Unfortunately, the National Cancer Registry provides only data regarding patients’ survival, while old information regarding primary tumors is not available. In summary, the results of the analysis should be interpreted with caution. Despite that, this study can provide valuable data on this important topic due to the relatively large sample size with this very rare entity.

Due to the rarity of RIS and lack of guidelines, it would be highly advisable for patients to participate in clinical trials. However, access to sarcoma-dedicated trials could be limited by the frequent exclusion of patients with a history of RT in the affected area or history of second active malignancy with a required long, disease-free period. Thus, data collection in prospective registries may be an alternative approach.

## 5. Conclusions

Despite intensive treatment, the prognosis of RIS remains poor. Surgery with curative intent remains the basic method of RIS management. However, the optimal treatment regimen and the role of other modalities are not established. Secondary RT is rarely used in RIS. The development of new clinical trials or prospective registries should be encouraged.

## Figures and Tables

**Table 1:** Primary tumor characteristics.

Characteristic		Value
Age at Primary Radiotherapy	Median (Range)	43 (3–76) Years
		Number of enrolled patients (%)
Sex	Male	43 (74)
	Female	15 (26)
Received treatment	Radiotherapy	35 (60)
	Radiochemotherapy	25 (40)
Primary cancer	Breast cancer	20 (35)
	Sarcoma	11 (19)
	Other	10 (17)
	Hodgkin lymphoma	8 (14)
	Uterine cancer	5 (9)
	Non-Hodgkin lymphoma	4 (7)
Primary site (most of irradiated volume)	Thorax	19 (33)
	Pelvis	18 (31)
	Breast	11 (19)
	Head and neck	4 (7)
	Central nervous system	2 (3)
	Upper extremity	2 (3)
	Lower extremity	2 (3)

**Table 2:** Radiation-induced sarcoma characteristics.

Characteristic		Value
Age at RIS Diagnosis	Median (Range)	57 (20–84) Years
Years from primary RT to RIS diagnosis	Median (range)	11 (3–36) years
		Number of enrolled patients (%)
RIS site	Thorax	22 (38)
	Pelvis	15 (26)
	Breast	5 (9)
	Head and neck	4 (7)
	Upper extremity	4 (7)
	Abdominal cavity	3 (5)
	Lower extremity	3 (5)
	Central nervous system	2 (3)
RIS pathology	UPS	14 (24)
	Sarcoma NOS	12 (21)
	Osteosarcoma	9 (16)
	Angiosarcoma	7 (12)
	Fibrosarcoma and myxofibrosarcoma	6 (10)
	MPNST	4 (7)
	Leiomyosarcoma of the bone	1 (2)
	Chondrosarcoma	1 (2)
	Malignant GCT of the bone	1 (2)
	Synovial sarcoma	1 (2)
	Pleomorphic liposarcoma	1 (2)
	Round cell sarcoma	1 (2)
RIS grade	1	3 (5)
	2	16 (28)
	3	39 (67)

GCT—giant cell tumor; MPNST—malignant peripheral nerve sheath tumor; NOS—not otherwise specified; RIS—radiation induced sarcoma; RT—radiotherapy; UPS—undifferentiated pleomorphic sarcoma.

**Table 3:** Hazard ratios for death with 95% confidence intervals and *p*-values calculated from a univariate Cox proportional hazards model for all enrolled patients.

		Number of Cases	Hazard Ratio	95% Confidence Interval	*p*-Value
Primary treatment	radiotherapy	35	1		
	radiochemotherapy	23	1.16	0.58–2.32	0.675
RIS site	central nervous system	2	1		
	head and neck	4	0.19	0.03–1.40	0.104
	thorax	22	0.29	0.06–1.33	0.111
	breast	5	0.09	0.01–1.00	0.050
	upper extremity	4	0.06	0.01–0.71	0.026
	abdominal cavity	3	0.62	0.09–4.54	0.641
	pelvis	15	0.36	0.08–1.70	0.196
	lower extremity	3	0.68	0.09–4.84	0.697
RIS pathology	osteosarcoma	9	1		
	NOS	12	1.67	0.54–5.15	0.375
	UPS	14	1.43	0.44–4.64	0.550
	angiosarcoma	7	1.03	0.24–4.50	0.966
	myxo/fibrosarcoma	6	1.01	0.23–4.39	0.986
	MPNST	4	1.30	0.34–4.95	0.703
	other	6	1.07	0.25–4.52	0.926
Grade	1	3	1		
	2	16	1.62	0.20–13.01	0.652
	3	39	2.27	0.30–16.88	0.424
Locally curative treatment	no	18	1		
	yes	40	0.21	0.10–0.44	<0.001

MPNST—malignant peripheral nerve sheath tumor; NOS—sarcoma not otherwise specified; RIS—radiation-induced sarcoma; UPS—undifferentiated pleomorphic sarcoma.

**Table 4:** Hazard ratios for death with 95% confidence intervals and *p*-values calculated from a univariate Cox proportional hazards model for patients who underwent curative surgery.

		Number of Cases	Hazard Ratio	95% Confidence Interval	*p*-Value
Perioperative radiotherapy	no	29	1		
	yes	10	1.31	0.51–3.36	0.568
Perioperative chemotherapy	no	16	1		
	yes	23	0.86	0.34–2.15	0.742
Surgical margin	R0	18	1		
	R1	14	1.14	0.39–3.28	0.813
	R2	1	4.12	0.47–36.04	0.201
	unknown	6	2.71	0.79–9.35	0.114

R0—microscopically negative margins; R1—microscopically positive margins; R2—gross residual disease.

## Data Availability

All data generated or analyzed during this study are available upon reasonable request.

## Supplementary Materials

The following are available online at https://www.mdpi.com/2077-0383/10/4/694/s1, Figure S1: Dose distribution of summed plan (reirradiation, transverse view), Figure S2: Dose distribution of summed plan (reirradiation, frontal view).
