# Supplementary material for: The Management of Radiation-Induced Sarcomas: A Cohort Analysis from a Sarcoma Tertiary Center
Source: J Clin Med. 2021 Feb 10;10(4):694. doi: 10.3390/jcm10040694 (PMC7916641; doi:10.3390/jcm10040694)
Supplement: Supplementary file 1 [file jcm-10-00694-s001.zip › Supplement_1_proof.pptx]

## Slide 1
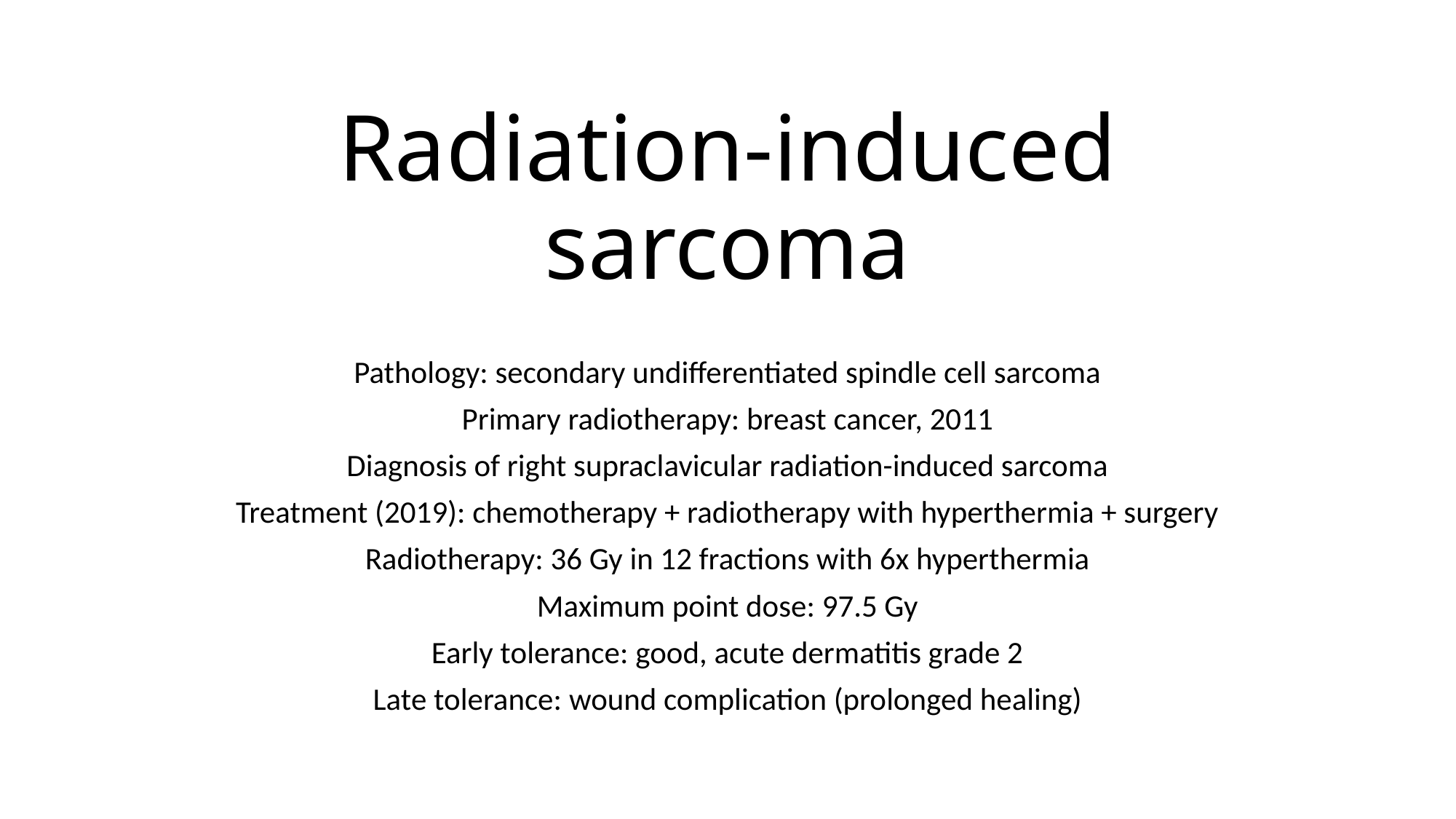

# Radiation-induced sarcoma
Pathology: secondary undifferentiated spindle cell sarcoma
Primary radiotherapy: breast cancer, 2011
Diagnosis of right supraclavicular radiation-induced sarcoma
Treatment (2019): chemotherapy + radiotherapy with hyperthermia + surgery
Radiotherapy: 36 Gy in 12 fractions with 6x hyperthermia
Maximum point dose: 97.5 Gy
Early tolerance: good, acute dermatitis grade 2
Late tolerance: wound complication (prolonged healing)

## Slide 2
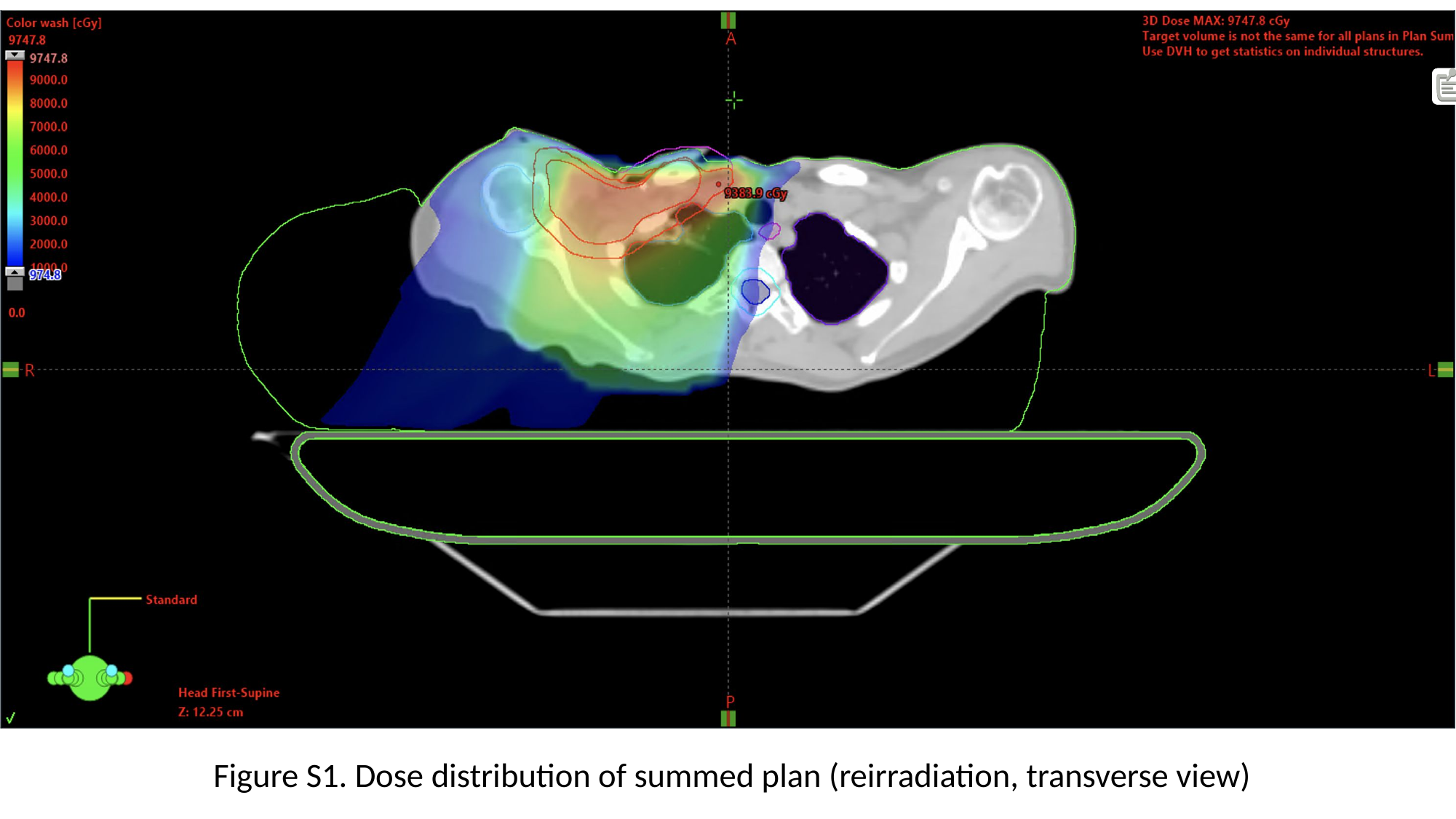

Figure S1. Dose distribution of summed plan (reirradiation, transverse view)

## Slide 3
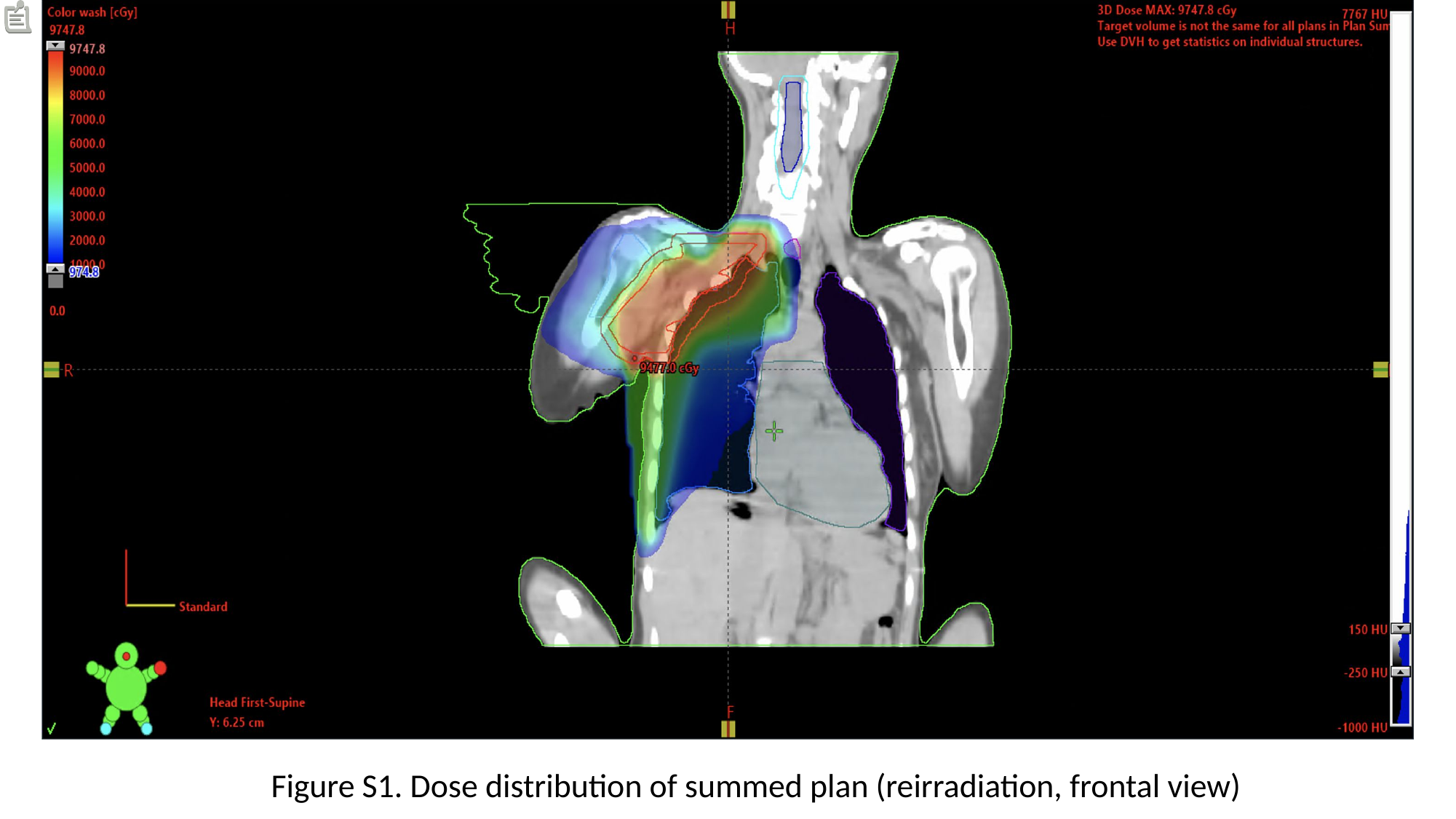

Figure S1. Dose distribution of summed plan (reirradiation, frontal view)
